# Methanolic Extract of *Ganoderma lucidum* Induces Autophagy of AGS Human Gastric Tumor Cells

**DOI:** 10.3390/molecules201017872

**Published:** 2015-09-29

**Authors:** Filipa S. Reis, Raquel T. Lima, Patricia Morales, Isabel C. F. R. Ferreira, M. Helena Vasconcelos

**Affiliations:** 1 I3S—Instituto de Investigação e Inovação em Saúde, Universidade do Porto, Porto 4099-002, Portugal; E-Mails: freis@ipatimup.pt (F.S.R.); rlima@ipatimup.pt (R.T.L.); 2 Cancer Drug Resistance Group, Institute of Molecular Pathology and Immunology of the University of Porto (IPATIMUP), Rua Júlio Amaral de Carvalho, 45, Porto 4200-135, Portugal; 3 Departamento de Nutrición y Bromatología II, Facultad de Farmacia, Universidad Complutense de Madrid (UCM), Pza Ramón y Cajal, s/n, E-28040 Madrid, Spain; E-Mail: patricia.morales@farm.ucm.es; 4 Mountain Research Center (CIMO), ESA, Polytechnic Institute of Bragança, Apartado 1172, Bragança 5301-855, Portugal; 5 Department of Pathology and Oncology, Faculty of Medicine, University of Porto, Alameda Professor Hernâni Monteiro, Porto 4200-319, Portugal; 6 Laboratory of Microbiology, Department of Biological Sciences, Faculty of Pharmacy, University of Porto, Rua de Jorge Viterbo Ferreira n.° 228, Porto 4050-313, Portugal

**Keywords:** *Ganoderma lucidum*, methanolic extract, antitumor potential, programmed cell death (PCD), autophagy

## Abstract

*Ganoderma lucidum* is one of the most widely studied mushroom species, particularly in what concerns its medicinal properties. Previous studies (including those from some of us) have shown some evidence that the methanolic extract of *G. lucidum* affects cellular autophagy. However, it was not known if it induces autophagy or decreases the autophagic flux. The treatment of a gastric adenocarcinoma cell line (AGS) with the mushroom extract increased the formation of autophagosomes (vacuoles typical from autophagy). Moreover, the cellular levels of LC3-II were also increased, and the cellular levels of p62 decreased, confirming that the extract affects cellular autophagy. Treating the cells with the extract together with lysossomal protease inhibitors, the cellular levels of LC3-II and p62 increased. The results obtained proved that, in AGS cells, the methanolic extract of *G. lucidum* causes an induction of autophagy, rather than a reduction in the autophagic flux. To our knowledge, this is the first study proving that statement.

## 1. Introduction

One of the hallmarks of cancer is the ability of tumor cells to resist cell death [1]. Therefore, therapies based on the induction of programmed cell death (PCD) constitute an area of scientific interest.

Being a “self-digestive” process, autophagy is considered a mechanism of cell survival, which allows the cell to obtain nutrients under deprivation conditions [2]. However, recent investigations have shown that, despite its importance as a homeostatic evolutionary process in all cells, autophagy may also be considered a mechanism of cell death. Actually, cells exposed to prolonged stress and continuous autophagy can eventually “commit suicide” [3].

Mushrooms are a proven source of extracts and/or compounds with antitumor properties, *namely* with capacity to induce PCD [4,5,6]. Thus, the identification and chemical characterization of mushroom extracts that induce PCD in human tumor cells may allow the discovery of novel compounds that can be used as drugs or nutraceuticals.

*Ganoderma lucidum* (Curtis) P. Karst. has been used as a functional food and as preventive medicine in the Far East and has become a popular dietary supplement in Western countries. This mushroom has been extensively reported as having important bioactive properties, including antitumor activity via induction of programmed cell death (PCD) [7,8,9]. Moreover, some of the *G. lucidum* extracts and/or isolated compounds have been previously described as modulators of autophagy in several human tumor cell lines [10,11,12,13]. Indeed, some of us have previously report that a methanolic extract of *G. lucidum* fruiting body (obtained by extraction at room temperature) inhibited the growth of a human gastric tumor cell line, through a mechanism which involved cellular autophagy [13]. However, it is not known if the extract is an inducer of autophagy or an inhibitor of the autophagic flux.

In the present work we aimed at studying a presumably more potent methanolic extract from *G. lucidum*, obtained by cold extraction, in order to understand if the previously observed autophagy modulation was due to an increase in autophagy or to a decrease in the late stages of autophagy (*i.e.*, a block in the autophagic flux). Moreover, the phenolic content of the extract was determined and cellular treatments with the phenolics found in the extract were performed, in order to verify if they were responsible for the extract bioactivity.

## 2. Results and Discussion

### 2.1. Chemical Characterization of the Methanolic Extract

**Figure 1:**
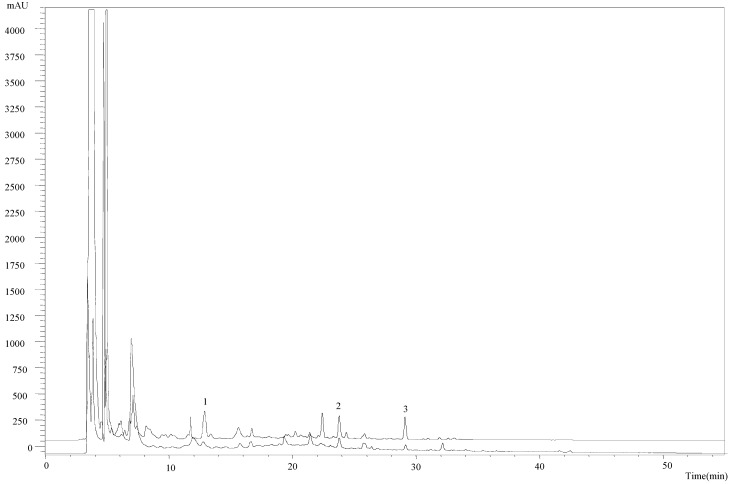
Phenolic acids profile of *Ganoderma lucidum* cold extract (-----) and room temperature extract (^_____^). 1—*p*-Hydroxybenzoic acid; 2—*p*-coumaric acid; 3—cinnamic acid.

The methanolic extract obtained by cold extraction was chemically characterized, revealing the presence of *p*-hydroxybenzoic acid (123 ± 9 µg/g extract), *p*-coumaric acid (80 ± 6 µg/g extract) and cinnamic acid (59 ± 6 µg/g extract). Compared with the profile of the methanolic extract obtained at room temperature: *p*-hydroxybenzoic acid (82 ± 4 µg/g extract), *p*-coumaric acid (53 ± 3 µg/g extract) and cinnamic acid (40 ± 3 µg/g extract) [13], the former revealed a higher concentration of all phenolic and related compounds (Figure 1).

### 2.2. Antitumor Potential of the New Obtained Extract

In order to confirm if the cold extract presented antitumor activity, the new extract was tested against a panel of four human tumor cell lines (Table 1).

Even though the studies were not carried out at the same time, the cold methanolic extract here studied presented a more potent *in vitro* cell growth inhibitory activity than the previously studied extract (prepared at room temperature), under the same laboratorial conditions and using the same cell lines [13]. This could be attributed to the higher content of phenolic acids and related compounds observed in the cold extract. Therefore, the following study was performed with this extract of the *G. lucidum* fruiting body and in the AGS cell line.

**Table 1:** GI_50_ concentrations of the cold methanolic extract of *G. lucidum* fruiting body in various human tumor cell lines ^a^.

*Ganoderma lucidum* Methanolic Extract				
GI_50_ (µg/mL)	NCI-H460	HCT-15	MCF-7	AGS
	77.9 ± 3.3	70.0 ± 2.4	67.2 ± 3.1	66.6 ± 4.4

^a^ Values correspond to the mean ± SE of at least three independent experiments. The maximum DMSO concentration used was 0.3% and did not interfere with cell growth (% cell growth relatively to Blank cells treated with medium was as follows: 115.7% ± 0.12% in AGS cells, 108.6% ± 0.07% in NCI-H460 cells, 99.2% ± 0.04% in HCT-15 cells and 106.9% ± 0.05% in MCF-7 cells). Previously published GI_50_ results with an extract obtained at room temperature were as follows: 107.5 ± 5.3 µg/mL in NCI-H460 cells, 103.4 ± 13.2 µg/mL in HCT-15 cells, 112.6 ± 6.7 µg/mL in MCF-7 cells and 93.3 ± 9.1 µg/mL in AGS cells [13].

### 2.3. Effect of G. lucidum Methanolic Extract in AGS Cellular Autophagy

**Figure 2:**
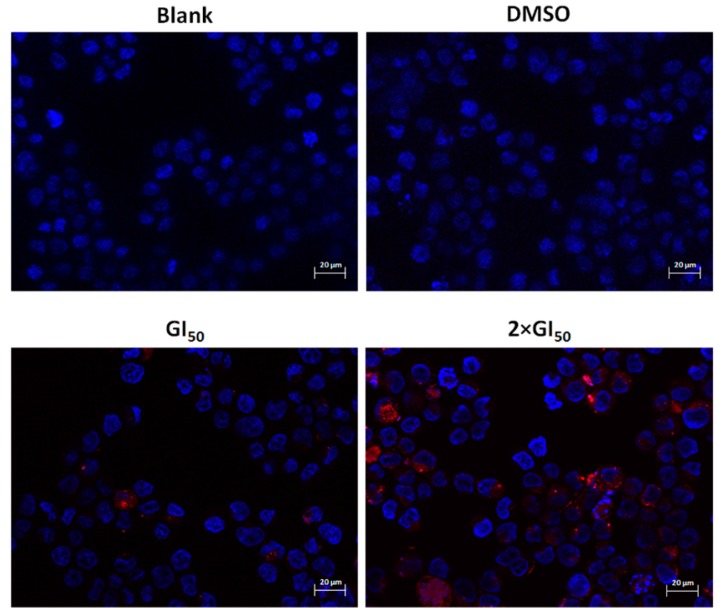
Analysis of the presence of autophagic vacuoles following treatment with *G. lucidum* methanolic extract, by fluorescence microscopy. Cells were transfected for 24 h with LC3 mCherry vector (**red**) and further treated for 48 h with: medium only (**Blank**), with the methanolic extract (at GI_50_ and 2 × GI_50_ concentrations) or with the extract solvent (DMSO) corresponding to the higher concentration of the extract. Nuclei were stained with DAPI (**blue**). Images are representative of two experiments. Bar corresponds to 20 μm.

AGS cells were transfected with a mCherry-LC3 expression vector and were then treated for 48 h with two concentrations of the extract (GI_50_ and 2 × GI_50_ concentrations). These concentrations were selected since they inhibited the growth of the cell population but did not cause much cell death (Supplementary Material). The obtained results indicate that this treatment induced the presence of LC3 within autophagic vacuoles (Figure 2).

This effect was preferentially observed in cells treated with a concentration of 133.2 µg/mL of the extract. The presence of autophagic vacuoles (autophagosomes), typical of autophagy, was thus confirmed in AGS cells following treatment with the extract.

**Figure 3:**
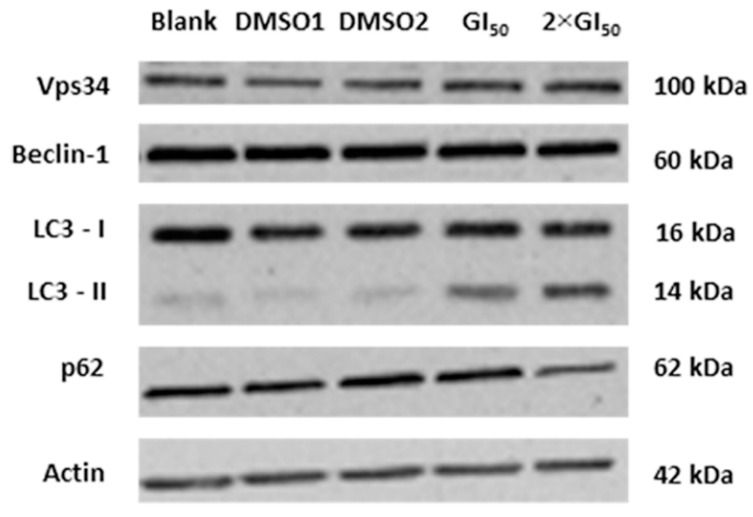
Analysis of the levels of the autophagy-related proteins Vps34, Beclin-1, LC3-I, LC3-II and p62, following treatment with *G. lucidum* methanolic extract, analyzed by Western blot. Cells were treated for 48 h with: medium only (Blank), with the methanolic extract (at GI_50_ and 2 × GI_50_ concentrations) or the corresponding volumes of the extract solvent (DMSO). Actin was used as loading control. Image is representative of three independent experiments.

It is known that Beclin-1 binds to the vacuolar sorting protein 34 (Vps34, a class III phosphatidylinositol-3 kinase), and that this complex is involved in the initial step of autophagosomes formation [14]. Therefore, the expression levels of such proteins in AGS cells following 48 h treatment with the two tested concentrations of the extract were investigated. Nevertheless, no differences in the expression levels of these proteins were found at this time point (Figure 3).

In addition, the levels of other autophagy-related proteins (LC3 and p62) were analyzed following the same treatment. LC3 turnover is usually monitored to follow the autophagic flux. This is based on the measurement of the levels of LC3-II, which are known to increase upon autophagy induction and decrease thereafter (since it is degraded in the autolysosomes) [15,16]. Thus, an increase in LC3-II protein levels is expected to be found at the beginning of the autophagy mechanism, as this protein is associated with autophagosomes formation. On the other hand, in the course of the autophagic flow a decline in these levels is expected, since the autophagic vacuoles are ultimately degraded. Furthermore, p62 (another autophagy substrate) can also be used to monitor the autophagic flux. This protein directly interacts with LC3 and, as the LC3-II present in the inner membrane of the autophagosomes is degraded by lysosomal proteases, the p62 previously trapped by LC3 is selectively incorporated into autophagosomes and is degraded by autophagy. Accordingly, the total cellular levels of p62 correlate inversely with autophagic activity and a decrease in p62 levels is expected with the induction of autophagy [15,17]. Results showed that this treatment caused an increase in the cellular levels of LC3-II, together with a slight reduction in the levels of p62, particularly with the highest concentration studied (Figure 3). These results are in agreement with an apparent induction of autophagy. However, the observed results could be due to an induction of autophagy or to a decrease of the autophagic flux. To confirm this, the levels of LC3-II and p62 were further analyzed following cellular treatment with the extract together with (or without) the lysossomal protease inhibitors of the formation of the autophagolysosome (E-64d/pepstatin, known later-stage autophagy inhibitors) [18].

**Figure 4:**
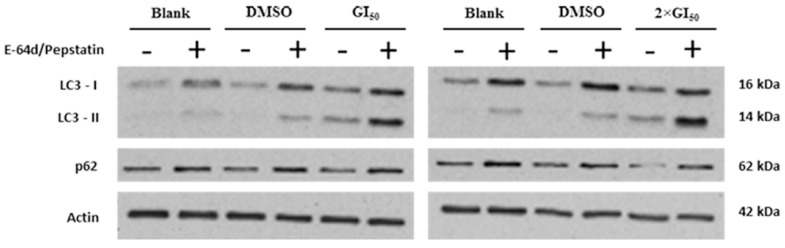
Effect of co-treatment with *G. lucidum* methanolic extract together with E-64d/pepstatin in LC3-II and p62 levels, analyzed by Western blot. Cells were treated for 48 h with medium (Blank), with the solvent (DMSO) or with the extract (GI_50_ and 2 × GI_50_ concentrations), together with (or without) E-64d/pepstatin (15 µg/mL). Actin was used as loading control. Results are representative of 3 independent experiments.

Results showed that the levels of both LC3-II and p62 proteins increased following treatment of cells with the extract together with the inhibitors, when compared to the treatment with the extract alone (Figure 4). These results confirm that this extract of *G. lucidum* is an inducer of autophagy.

The presented results further support the fact that the methanolic extract of *G. lucidum* may be used as a source of compounds which induce autophagy.

**Figure 5:**
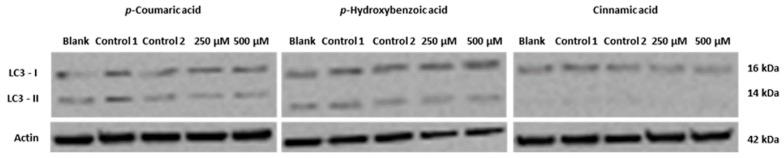
Analysis of the levels of the autophagy marker LC3II in AGS cells following 48 h treatment with *p*-coumaric acid, *p*-hydroxybenzoic acid or cinnamic acid, analyzed by Western blot. Cells were treated for 48 h with medium (Blank), with the solvent (DMSO:water; 1:1 *v*/*v*; controls 1 and 2) or with the compounds (at 250 and 500 µM). Actin was used as loading control. Blot is representative of two independent experiments.

Since some phenolic acids were identified in the methanolic extract of *G. lucidum*, namely *p*-coumaric and *p*-hydroxybenzoic acids and the related compound cinnamic acid, we also tried to assess whether these compounds were responsible for the studied bioactivity. The levels of the autophagy marker LC3-II were analyzed in AGS cells following treatment with relatively high concentrations of these compounds (250 µM and 500 µM), but no significant alterations were observed (Figure 5), indicating that these compounds are probably not involved, at least not on their own, in the induction of autophagy that was observed in cells treated with the extract.

Additionally, we tried to verify if some derivatives of *G. lucidum* phenolic acids (synthesized from the main phenolic acids present in the extract—*p*-hydroxybenzoic and cinnamic acids; Heleno *et al.* [19]) were inducers of autophagy. However, none of the synthesized compounds tested (methylated and acetyl glucuronated methyl esters derivatives) were capable of increasing the cellular LC3-II levels, up to the concentrations tested (20, 40, and 60 µM, data not shown). Nevertheless, it’s possible that the phenolic compounds may act synergistically with other compounds. This concomitant effect has been proven both *in vitro* and *in vivo* [20,21,22,23,24], and may be a possible justification for the results obtained.

## 3. Experimental Section

### 3.1. Collection and Sample Preparation

Wild samples of *Ganoderma lucidum* (Curtis) P. Karst. were harvested in Bragança (Northeastern Portugal) in July 2011. The taxonomic identification and sample preparation was made as described by Heleno *et al.* [25].

### 3.2. Preparation and Chemical Characterization of Ganoderma lucidum Methanolic Extract

A cold methanolic extraction was applied to *G. lucidum* fruiting bodies, by mixing the dry powder (~1 g) with methanol (30 mL) at −20 °C (freezer) for 2 h. After sonication for 15 min, the extract was filtered and the residue was then extracted with two additional 30 mL portions of methanol; combined extracts were concentrated in a rotavapor. The obtained extract was chromatographically characterized according to other authors [25].

To the accomplishment of biological assays, the residue extracts were re-dissolved in DMSO (100 mg/mL).

### 3.3. Screening of the Growth Inhibitory Activity in Human Tumor Cell Lines

The effects of the *G. lucidum* methanolic extract on the growth of human tumor cell lines were evaluated with the sulforhodamine B (SRB) assay [26]. Four human tumor cell lines were used: AGS (gastric adenocarcinoma), MCF-7 (breast adenocarcinoma), NCI-H460 (non-small cell lung cancer) and HCT-15 (colorectal adenocarcinoma), and the screening was performed as already described by Oliveira *et al.* [13].

### 3.4. Transfection with LC3-mCherry Expression Vector

Cells were plated in 24-well plates (3.75 × 10^4^ cells/well) and allowed to adhere for 24 h. Transfection with the vector LC3-mCherry (a kind gift from Prof. T. Johansen) [27] was then carried out using Lipofectamine (Invitrogen) according to manufacturer’s instructions. As described by Birame *et al.* [28] during the initial 4 h of transfection, cells were incubated with medium containing 5% FBS, which was then replaced by medium containing 10% FBS. Following 24 h of transfection, medium was removed and cells were treated for 48 h with medium (Blank); with the studied methanolic extract from *G. lucidum* (at GI_50_ concentration of 66.6 µg/mL, and 2 × GI_50_ concentration of 133.2 µg/mL, as previously determined with the SRB assay, Table 1); or with the extract solvent (DMSO) corresponding to the higher concentration. Cells were then fixed in 4% paraformaldehyde in PBS and analyzed in a fluorescence microscope (Axio Imager.Z1 coupled with ApoTome Imaging System microscope, Zeiss, Oberkochen, Germany).

### 3.5. Expression of Autophagy-Related Proteins

AGS cells were plated at 1.5 × 10^5^ cells/well in 6-well plates and incubated for 24 h. Cells were then treated with the methanolic extract of *G. lucidum* at its GI_50_ and 2 × GI_50_ concentrations. Blank cells (treated with medium) or control cells (treated with DMSO, corresponding to the GI_50_ and 2 × GI_50_ concentrations of the extract) were also included. For treatments with the phenolic acids and related compounds, cells were treated with *p*-coumaric, *p*-hydroxybenzoic and cinnamic acids for 48 h with concentrations of 250 and 500 µM (parental compounds) and 20, 40, and 60 µM (methylated and acetyl glucuronated methyl esters derivatives). Blank and control cells were also included. 

Following 48 h of treatment, AGS cells were lysed and protein lysates were quantified according to manufacturer’s instructions and 20 µg of protein loaded on 12% SDS-PAGE gel [29]. After electrophoretic transfer into nitrocellulose membranes (GE Healthcare, Oeiras, Portugal), membranes were incubated with the following primary antibodies: rabbit anti-VPS34 (1:1000, Cell Signaling, Danvers, MA, USA), rabbit Beclin-1 (1:1000, Cell Signaling), rabbit anti-Light Chain 3 B, LC3 (1:1000, Cell Signaling), rabbit anti-p62 (1:5000, Enzo, New York, NY, USA), goat anti-Actin antibody (1:2000, Santa Cruz Biotechnology, Heidelberg, Germany) and with the corresponding secondary antibody: goat anti-rabbit IgG-HRP (1:2000, Santa Cruz Biotechnology), donkey anti-goat IgG-HRP (1:2000, Santa Cruz Biotechnology) or goat anti-mouse IgG-HRP (1:2000, Santa Cruz Biotechnology). Signal was detected using Amersham™ ECL Western Blotting Detection Reagents (GE Healthcare), the Amersham Hyperfilm ECL (GE Healthcare) and the Kodak GBX developer and fixer (Sigma-Aldrich, Sintra, Portugal).

### 3.6. Treatment with Autophagy Inhibitors E-64d/Pepstatin

AGS cells were plated in 6-well plates (1.5 × 10^5^ cells/well) and allowed to adhere for 24 h. Cells were then treated for 1 h with 15 µg/mL of the lysossomal inhibitors E-64d (AppliChem, Darmstadt, Germany) and Pepstatin A (Cayman Chemical, Ann Arbor, MI, USA) and then co-incubated for 48 h with the GI_50_ and 2 × GI_50_ concentrations of the studied methanolic extract. Blank and control cells were also included. Protein expression analysis was carried out by Western blot, as described above.

### 3.7. Statistical Analysis

All experimental data are presented as mean values ± standard deviation (SD) or standard error (SE) from at least three independent experiments (most of them performed in duplicate).

## 4. Conclusions

In conclusion, *G. lucidum* methanolic extract increased the formation of autophagosomes, increased the cellular levels of LC3-II and decreased the cellular levels of p62, confirming that the extract affects cellular autophagy in AGS cells. Additionally, treatment with the extract together with lysossomal protease inhibitors caused a further increase in the cellular LC3-II levels together with an increase in p62 levels proving that, in these cells, the methanolic extract of *G. lucidum* cause an induction of autophagy rather than a reduction in the autophagic flux. Further studies are still needed to identify the bioactive compound(s) present in the tested extract which may be responsible for the observed autophagy induction. Moreover, future work will allow elucidating which are the intracellular signaling cascades involved in autophagy induced by *G. lucidum* or by their bioactive compounds, namely the PI3K/mTOR or the AMPK signaling pathways.

## Supplementary Materials

Supplementary materials can be accessed at: http://www.mdpi.com/1420-3049/20/10/17872/s1.
