# Supplementary material for: Methanolic Extract of Ganoderma lucidum Induces Autophagy of AGS Human Gastric Tumor Cells
Source: Molecules. 2015 Sep 29;20(10):17872–82. doi: 10.3390/molecules201017872 (PMC6332321; doi:10.3390/molecules201017872)
Supplement: Supplementary file 1 [file molecules-20-17872-s001.pdf]

Supplementary Material

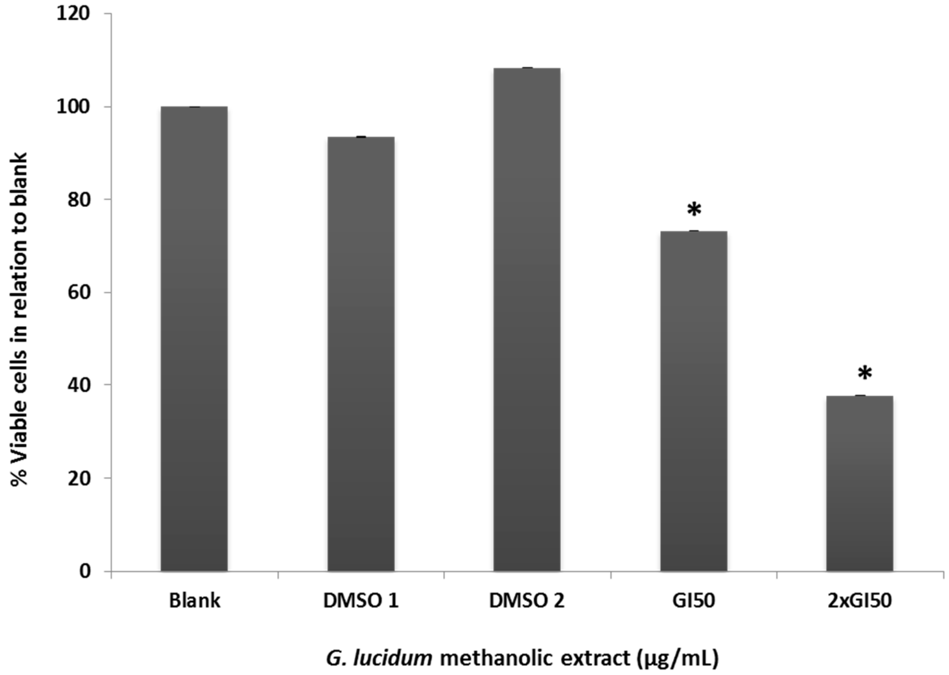

Figure S1. Effect of *G. lucidum* methanolic extract on AGS viable cell number. Results were analysed 48 h after incubation with medium only (Blank), with the methanolic extract (at GI_{50} and $2 \times GI_{50}$ concentrations) or the corresponding volumes of the extract solvent (DMSO). Results are presented as a percentage of viable cells in relation to blank cells and are the mean ± SE of six independent experiments. * $p < 0.05$ blank *vs*. treatment.
